# Effects of *GSTM1* in Rheumatoid Arthritis; Results from the Swedish EIRA study

**DOI:** 10.1371/journal.pone.0017880

**Published:** 2011-03-22

**Authors:** Emeli Lundström, Toinette Hartshorne, Kelly Li, Staffan Lindblad, Marius C. Wick, Camilla Bengtsson, Lars Alfredsson, Lars Klareskog, Leonid Padyukov

**Affiliations:** 1 Department of Rheumatology, Karolinska Institutet, Stockholm, Sweden; 2 Applied Biosystems, Foster City, California, United States of America; 3 Department of Radiology, Innsbruck Medical University, Innsbruck, Austria; 4 Institute of Environmental Medicine, Karolinska Institutet, Stockholm, Sweden; Sheba Medical Center, Israel

## Abstract

**Objective:**

Glutathione-*S*-transferases (GSTs) play an important role in tobacco smoke detoxification, interestingly approximately 50% of individuals in most human populations lack the gene *GSTM1* due to copy number variation (CNV). We aimed to investigate *GSTM1* CNV in Rheumatoid Arthritis (RA) in relation to smoking and *HLA*-DRB1 shared epitope; the two best known risk factors for RA and in addition, to perform subanalyses in patients where relations between variations in *GSTM1* and RA have previously been described.

**Methods:**

qPCR was performed using TaqMan Copy Number assays (Applied Biosystems) for 2426 incident RA cases and 1257 controls from the Swedish EIRA. Odds ratio (OR) together with 95% confidence intervals (CI) was calculated and used as a measure of the relative risk of developing RA.

**Results:**

No association between RA and *GSTM1* CNV was observed when analyzing whole EIRA. However, ≥1 copy of *GSTM1* appears to be a significant risk factor for autoantibody positive RA in non-smoking females ≥60 years (OR: 2.00 95% CI: 1.07–3.74), a population where such relationships have previously been described. Our data further suggest a protective effect of *GSTM1* in ACPA-negative smoking men (OR: 0.56 95% CI: 0.35–0.90).

**Conclusion:**

We assessed the exact number of *GSTM1* gene copies in relation to development and severity of RA. Our data provide support for the notion that variations in copy numbers of *GSTM1* may influence risk in certain subsets of RA, but do not support a role for *GSTM1* CNV as a factor that more generally modifies the influence of smoking on RA.

## Introduction

Rheumatoid arthritis (RA) is characterized by chronic inflammation of synovial joints, resulting in progressive destruction of cartilage and bone. Increasing evidence exist that genes and environment interact in the development of the disease, and also that two major subsets of RA exists defined by presence/absence of antibodies to citrullinated protein antigens (ACPAs) [1]. In particular, a smoking history together with the shared epitope alleles (SE) of the *HLA*-DRB1 locus in the major histocompatibility complex have repeatedly been found to strongly increase the risk of developing RA, in ACPA-positive RA [2], [3], [4], [5], [6], [7].

A central feature of RA is inflammation with the resulting reactive oxygen species (ROS), which causes oxidation of macromolecules giving rise to a variety of cytotoxic products. ROS are produced by phagocytes in the synovial fluid and pannus and by synovial endothelial cells during hypoxia-reperfusion events. This means that variations in host effectiveness in detoxification of products of ROS activity might be important and there is growing evidence that ROS and their by-products may play a direct role in the development of RA [8], [9]. An alternative view on oxidative mechanisms was developed when up-regulation of *NcfI* in a rat model for arthritis was shown to be protective [10]. These data indicate that genetic differences in oxidative events may modulate the risk and/or severity of RA, and that several mechanisms may be involved, some even working in opposite directions. Since genetic polymorphisms of enzymes such as glutathione *S*-transferases (GSTs) have been shown to have an important role in detoxifying foreign substances from tobacco smoke, influencing susceptibility to both lung and colon cancer [11], [12] it is tempting to speculate that such polymorphisms may be of importance also for the development of RA, and that such influences may be very complex [13].

Glutathione *S*-transferase M1 (*GSTM1*) a member of the GST μ class has been localized to human chromosome 1p13.3 and is polymorphic in humans. Approximately 50% of individuals in most human populations completely lack the gene and thus the activity of *GSTM1* [14], [15]. The deletion of the *GSTM1* gene seems to be caused by an unequal crossing over event between sequences about 5 kb downstream from *GSTM2* and *GSTM1*, which results in the deletion of the entire *GSTM1* gene [14]. Mammalian cells constitutively express a number of detoxifying enzymes such as the GST family. The GSTs, which are a superfamily of polymorphic enzymes, play an important role in detoxification by catalyzing the conjugation of many hydrophobic and electrophilic compounds with reduced glutathione [8]. Protection against a broad range of compounds including carcinogens, pesticides, antitumor agents and environmental pollutants has been suggested to be mediated in part by GSTs (OMIM 138350).

The *GSTM1* copy number variation (CNV) is in a sense unique and provides us with “natural knockouts” for this gene in human population. It has been difficult however to detect the exact number of gene copies and to discriminate the common state with one copy from two copies. The absolute majority of previous studies of *GSTM1* genetics were able only to detect “null” allele against “positive” and there are still no studies published which have addressed susceptibility to RA in relation to the exact number of copies of *GSTM1*.

Mattey et al., demonstrated in 1999 [16], that GSTM1 null RA patients had more severe radiological progression (higher Larsen score) independent of the effect of the *HLA*-DRB1 associated shared epitope (SE). The same group later demonstrated that smoking was specifically tightly associated with severe RA in individuals who carried the *GSTM1*-null polymorphism [17]. Criswell et al. [18] described in 2006 the development of RA among older Caucasian women and found a positive association between *GSTM1*-null genotype and risk of disease. All these studies were performed in relatively small cohorts (Mattey et al. [16], 164 patients; Mattey et al., [17], 277 patients and 577 controls, Criswell et al. [18], 115 patients and 466 controls). In two other studies (Yun BR et al., [8]; Morinobu S et al, [19]) with 258 RA patients from Korea and 108 RA patients from Japan, risk of RA development was also suggested to be higher in individuals lacking the *GSTM1* gene. However, recently Keenan et al. [20] published a study using Nurses' Health Study (549 RA patients and controls) where no association was observed between presence/absence of *GSTM1*. Hence, the data have so far been ambiguous.

In the present study, we aimed to investigate whether there is any association between *GSTM1* CNV and development as well as severity of RA in a larger case-control study consisting of all together 3682 individuals, including 2426 RA patients, using a quantitative identification of the number of gene copies. We also analyzed the impact of *GSTM1* in RA in both the ACPA-positive and ACPA-negative subsets of the disease separately.

## Results

### GSTM1 CNV as a risk factor for RA

The *GSTM1* CNV frequencies in cases were 53.7% for 0 copies, 38.3% for 1 copy, 7.8% for 2 copies and 0.13% for 3 copies and in controls 51.8% for 0 copies, 40.1% for 1 copy, 7.9% for 2 copies and 0.08% for 3 copies. These frequencies are in line with what has been published previously concerning Caucasian populations [21], [22]. Our genotyping quality control demonstrated 100% match between commercial assay and our in-house PCR and the distribution of genotypes was in Hardy-Weinberg equilibrium.

We performed analysis using the EIRA study for association between *GSTM1* CNV and RA risk. Overall, no significant association was detected with development of RA, neither alone (OR: 0.93 95% CI: 0.81–1.06) or after stratification by smoking status (OR_ever smoker_: 0.92 95% CI: 0.76–1.11) (OR_never smoker_: 0.93 95% CI: 0.72–1.21) or by the SE alleles (OR_no SE_: 0.93 95% CI: 0.74–1.16) (OR_yes SE_: 0.91 95% CI: 0.76–1.09) (Table 1). We also compared individuals with no copies of *GSTM1* with individuals with ≥2 copies and found similar results; *GSTM1* CNV and development of RA OR: 0.95 95% CI: 0.74–1.24, smoking (OR_ever smoker_: 0.94 95% CI: 0.66–1.36) (OR_never smoker_: 1.02 95% CI: 0.64–1.65) and SE (OR_no SE_: 1.06 95% CI: 0.68–1.65) (OR_yes SE_: 0.84 95% CI: 0.60–1.17).

**Table 1:** Frequency of GSTM1 CNV in EIRA by ACPA status and different subgroups according to smoking and *HLA*-DRB1 status.

GSTM1 – RA overall				
GSTM1	Sex	Ca/co	OR	95% CI
No	All	1282/479	1,00	-
Any	All	1104/601	0,93	0.81–1.06
No	Women	908/465	1,00	-
Any	Women	792/425	0,95	0.81–1.12
No	Men	374/182	1,00	-
Any	Men	312/176	0,86	0.67–1.11

GSTM1 – ACPA-positive RA					GSTM1 – ACPA-negative RA		
GSTM1	Sex	Ca/co	OR	95% CI	Ca/co	OR	95% CI
No	All	670/639	1,00	-	407/639	1,00	-
Any	All	581/590	0,94	0.80–1.10	348/590	0,93	0.77–1.11
No	Women	474/457	1,00	-	279/457	1,00	-
Any	Women	412/419	0,95	0.79–1.14	260/419	1,02	0.82–1.26
No	Men	196/182	1,00	-	128/182	1,00	-
Any	Men	169/171	0,92	0.69–1.23	182/171	0,73	0.52–1.03

GSTM1 –RA among ever smokers					GSTM1 –RA among never smokers		
GSTM1	Sex	Ca/co	OR	95% CI	Ca/co	OR	95% CI
No	All	600/330	1,00	-	295/215	1,00	-
Any	All	530/318	0,92	0.76–1.11	259/202	0,93	0.72–1.21
No	Women	412/232	1,00	-	228/161	1,00	-
Any	Women	375/217	0,97	0.77–1.23	204/162	0,89	0.67–1.19
No	Men	188/98	1,00	-	67/54	1,00	-
Any	Men	155/101	0,80	0.56–1.14	55/40	1,11	0.64–1.91

GSTM1 –RA among SE-positives					GSTM1 –RA among SE-negatives		
GSTM1	Sex	Ca/co	OR	95% CI	Ca/co	OR	95% CI
No	All	950/325	1,00	-	328/299	1,00	-
Any	All	806/303	0,91	0.76–1.09	298/293	0,93	0.74–1.16
No	Women	669/244	1,00	-	236/205	1,00	-
Any	Women	568/215	0,96	0.78–1.19	224/206	0,94	0.72–1.23
No	Men	281/81	1,00	-	92/94	1,00	-
Any	Men	238/88	0,78	0.55–1.10	74/87	0,87	0.57–1.33

Ca/Co = Cases and controls.

OR = odds ratio.

95% CI = 95% confidence interval.

In addition to these data on *GSTM1*, our study confirms the previously known risk factors for ACPA-positive RA, *HLA*-DRB1 SE alleles (OR: 3.73 95% CI: 2.83–4.92) and smoking (OR: 1.74 95% CI: 1.35–2.25) and also their combination (OR: 8.00 95% CI: 4.96–12.89). Presence of *GSTM1* did not significantly influence this combined effect (among *GSTM1* positive, OR: 7.46 95% CI: 4.61–12.06) (Table 2).

**Table 2:** Influence of GSTM1 on development of ACPA-positive RA in relation to SE and smoking.

No GSTM1 – ACPA-positive RA				
SE	Sex	Ca/co	OR	95% CI
No	All	97/204	1.0	-
Any	All	571/322	3.73	2.83–4.92

No GSTM1 – ACPA-positive RA				
Smoking	Sex	Ca/co	OR	95% CI
Never	All	147/212	1.0	-
Ever	All	392/325	1.74	1.35–2.25

No/Yes GSTM1 – ACPA-positive RA				
SE, Ever smoker, No GSTM1	Sex	Ca/co	OR	95% CI
No SE, Never smoker, No GSTM1	All	25/97	1.0	-
Yes SE, Ever smoker, No GSTM1	All	338/164	8.0	4.96–12.89
Yes SE, Ever smoker, Yes GSTM1	All	296/154	7.46	4.61–12.06

Ca/Co = Cases and controls.

OR = odds ratio.

95% CI = 95% confidence interval.

### GSTM1 CNV in relation to RA severity

We performed analysis in our study to address to what degree *GSTM1* CNV associates with baseline disease activity (DAS28) and with erosions at baseline, 1 and 2 years after disease onset. We found no differences in DAS28 between individuals carrying 0, 1, 2 or 3 copies of *GSTM1* (p = 0.82) (Table 3). When studying X-ray data we observed that Larsen score at baseline in female ever smokers moderately associated to the gene copy number of *GSTM1* (p = 0.04) (Table 4). The influence from *GSTM1* was significant when analyzing presence of the gene vs. absence (p = 0.006) but did not remain significant when analyzing ≥2 vs. 0 copies (non-corrected p = 0.04). No association with *GSTM1* CNV was observed, however, when analyzing erosions in RA patients 1 or 2 years after disease onset.

**Table 3:** Frequency of GSTM1 CNV and severity of RA.

Covariate	Copies	Cases	Mean	Lower 95%–Upper 95%	p
DAS28					0.82
	0	755	4.99	4.90–5.09	
	1	524	5.06	4.94–5.18	
	2	107	4.96	4.70–5.22	
	3	3	4.99	3.43–6-56	
Larsen score, female smokers					0.04
	0	36	3.54	1.77–5.32	
	1	28	5.42	3.41–7.43	
	2	4	7.19	1.86–12.52	
	3	1	3	7.66–13.66	

DAS28 and Larsen score retrieved at baseline.

Non-parametric statistical test (Mann-Whitney).

**Table 4:** Presence of GSTM1 and risk of developing ACPA-positive and ACPA-negative RA in relation to gender and age.

GSTM1 – ACPA-negative RA, male smokers			
GSTM1	Ca/co	OR	95% CI
No	41/97	01.00	-
Any	74/98	00.56	0.35–0.90

GSTM1 – ACPA-positive RA, female ≥60 years			
GSTM1	Ca/co	OR	95% CI
No	120/116	1.00	-
Any	122/161	1.37	0.96–1.93
No SE, No GSTM1	17/73	1.00	-
No SE, Yes GSTM1	49/23	2.02	0.98–4.16
Never smokers, No GSTM1	25/71	1.00	-
Never smokers, Yes GSTM1	36/51	2.00	1.07–3.74
Never smokers, No SE, No GSTM1	10/19	1.00	-
Never smokers, No SE, Yes GSTM1	4/36	3.32	0.86–12.77

Ca/Co = Cases and controls.

OR = odds ratio.

95% CI = 95% confidence interval.

### GSTM1 CNV and RA risk in relation to age of onset of disease

To test a hypothesis about an effect of *GSTM1* in the development of RA in older women, which was previously suggested by Criswell et al. [18], we restricted our analyses to ACPA-positive RA among women ≥60 years (mean age 64 years). In our study we found an insignificant trend towards an association between presence of *GSTM1* and risk of ACPA positive RA in these subjects (OR: 1.37 95% CI: 0.96–1.93) (Table 4). When subdividing the subjects according to smoking status, we found that presence of *GSTM1* is significantly associated with risk for ACPA-positive RA among never smoking women ≥60 years old (OR: 2.00 95% CI: 1.07–3.74) and we also found a trend for association between presence of *GSTM1* and ACPA positive RA among SE-negatives (OR: 2.02 95% CI: 0.98–4.16) (Table 5). We noticed a similar, non-significant trend between presence of *GSTM1* and ACPA positive RA when analyzing never smokers, not carrying SE (OR: 3.32 95% CI: 0.86–12.77). Additionally, when stratifying for ACPA-status and gender we saw a protective effect from the presence of *GSTM1* in ACPA-negative male smokers (OR: 0.56 95% CI: 0.35–0.90) (Table 4). However, these latter results should be interpreted with caution due to rather small groups and may well reflect chance effect.

**Table 5:** Baseline characteristics of case and control study population.

	Controls	Cases
Number	1257	2426
Females (%)	71.3	71.2
ACPA-positive (%)	0.8	62
Mean age ± SD (years)	52.9±11.6	51.3±12.4

ACPA = anti-citrullinated protein antibody.

19 controls and 391 patients were missing ACPA status.

## Discussion

The major finding in our study is the identification of complex associations between *GSTM1* CNV and risk- and severity of RA. Presence of *GSTM1* was associated with an increased risk of ACPA-positive RA among non-smoking older women, while presence of *GSTM1* was associated with a decreased risk of ACPA-negative RA among male smokers. No association with disease, in relation to disease activity and radiological changes were found when analyzing the whole study population. Notably, we did not find any direct impact of *GSTM1* CNV on the associations between RA and the strongest risk factors for this disease, i.e. HLA SE alleles and smoking. Instead our results suggest that variations in copy numbers of GSTM1 may influence the risk for RA in ways that are largely independent of smoking and HLA-DR SE genes. Based on the EIRA study design, the group of patients who are female, non-smokers and do not carry HLA-DR SE alleles are, constitute only approximately 4% of the total RA population, and it thus seems that *GSTM1* CNV does not play a substantial role for the majority of RA patients.

The absence of *GSTM1* was previously found to be a risk factor for RA and a factor contributing to disease severity [16], [17]. Thus, our new findings do not confirm some of the previous studies in relation to possible effect of *GSTM1* CNV. There are several possible reasons for this lack of confirmation. First, our findings may be a result from a type II error. We find this unlikely however, since the power to detect an odds ratio in the order of 1.5 is relatively high in our study. In contrast, all previous studies were based on lower number of cases and are likely underpowered and may thus represent results of type I errors. Second, the difference from previous studies on *GSTM1* in RA may be due to dissimilarity between the study populations. EIRA is a population based case-control study where controls have been randomly selected and matched to the cases on sex, age and residential area minimizing the potential for selection bias and population stratification. Since most mentioned previously published studies were cross sectional with long lasting RA (median 11 years [16], <5 years [23], 7 years [19]) and our study was preformed in incident cases, differences in results may have occurred due to an influence of GSTM1 variations on disease development and thus on which cases were available for recruitment in a long-lasting RA population. Interestingly, the largest previous studies of genetic risk from *GSTM1* (n the Nurese Health Study; Keenan et al (19) was also based on population-based recruitment and was not sensitive to selection on long-time severity, and this study on 549 RA cases and controls, Keenan et al. [20] could not demonstrate any association between *GSTM1* and development of RA. Also a smaller study on 115 incident cases was not able to identify any association of CNV [18] with RA.

Previously there was no robust technique to detect difference between heterozygous and homozygous states for the non-null *GSTM1* genotype. In the current study the effect from 2 copies of the gene in comparison with null genotype was investigated for the first time. However, not even using this refined analytic method provided any association between GSTM1 CNV on risk for RA in general. However, an increased risk was seen in the subgroup of non-smoking women ≥60 years old (though with broader confidence interval), most likely due to reduced power in our analyses compared to when analyzing presence (≥1 copy) vs. absence (0 copies) of *GSTM1*. This gene dosage effect nevertheless strengthens the possibility that there is a real association between *GSTM1* CNV and risk for RA in this subset of older non-smoking women.

It was previously suggested by Mattey et al., [17] that deletion of the *GSTM1* gene may influences RA severity measured as Larsen scores. This study was performed in a group of smokers with RA. Our data demonstrated no difference in the general RA study population regarding *GSTM1* CNV and disease activity at onset of disease or on rate of joint destruction after 1 or 2 years from diagnosis. Some trend for an influence of GSTM1 CNV on the baseline Larsen scores in female smokers was seen, but in opposite direction to what was has been described previously. We observed that ever smokers with at least one copy of *GSTM1* had more erosions in comparison to patients without *GSTM1*. This difference in relation to previously published data again could be well due to differences in selection criteria and population heterogeneity between the British (16) and our study. The conclusion in this article originated from analysis of 80 non-smokers and 84 smokers from UK Caucasian population with RA. In a regression model for this study the GSTM1-null state together with smoking provided only nominal p-value for regression coefficient (0.03), while the coefficients related to RF and disease duration were more robust (0.002 and <0.0001). This made the whole regression model for Larsen scores highly significant, but likely mainly dependent on other contributors than *GSTM1*. Finally, a study of 213 RA patients from Slovenia did not identify any association between variations in GSTM1 and DAS28 levels at the time of inclusion and these data are thus also in line with our finding [23].

Criswell et al., [18], who studied 115 incident cases described an association between *GSTM1* null homozygosity and risk for RA in older Caucasian women. They also observed that cigarette smoking was a more important risk factor for RA among those who carry the *GSTM1* gene. In order to specifically see whether we could replicate the results from this study, we excluded subjects below 60 years of age and thus analyzed female cases with an average age of 64 years (338 ACPA-positive cases, 166 ACPA-negative cases and 281 controls). We found an increased risk of developing ACPA-positive RA in non-smoking older females carrying *GSTM1* (OR: 2.12 95% CI: 1.14–3.95) as well as a trend in older females lacking the SE (OR: 2.02 95% CI: 0.98–4.16), thereby partly confirming some of the findings from Criswell et al in our larger study population.

In conclusion, our results suggest that *GSTM1* presence may increase risk of developing RA, but this effect is different in different subgroups of RA and is modified by gender and age. For future studies, it would be interesting to see if these results would be replicated in other large Caucasian and non-Caucasian study populations. Since we found a trend towards an association in individuals who lack the SE alleles and as SE does not appear to be an important risk factor for RA among certain non-Caucasian populations such as African and Hispanic American groups, it would be particularly interesting to study such groups in relation to genetic risk from GSTM1 [24], [25]. In addition, genetic variation and epigenetic regulation of other enzymes involved in detoxification of products of smoking may be of high interest for understanding the mechanisms behind development of RA.

## Materials and Methods

### Ethics statement

#### 96–174 EIRA

The research project IRB no 96–174 was approved 29/08/96 by the former local institutional review board at the Karolinska Institute.

### Study population

This study was based on a case-control study, the Epidemiological Investigation of Rheumatoid Arthritis (EIRA). The material involves incident cases of RA from different parts of Sweden ((2426 incident cases (1727 females and 699 males) of RA and 1257 controls (897 females and 360 males)). Successful genotyping was performed for 2386 (98,4%) cases (1700 females and 686 males) and 1249 (99,4%) controls (891 females and 358 males). Within this material we had access to data on baseline disease activity (DAS28) in 1426 cases (998 females and 428 males). Distribution of age, gender and ACPA status is depicted in Table 5. The study was approved by the ethics committee at the Karolinska Institutet and by Regional Stockholm ethics committee.

### Definition of smoking status

Smoking status was defined according to the EIRA questionnaire, as described previously [26]. For each case, the year when the first symptoms of RA occurred was defined as the index-year and the same index-year was used for the corresponding control. Briefly, subjects who reported that they regularly smoked cigarettes during or before the index-year were defined as *ever smokers* and those who reported that they had never smoked tobacco before or during the index-year were defined as *never smokers*.

### ACPA

Detection of antibodies to citrulline-containing peptides was performed using the Immunoscan-RA Mark2 ELISA test (Euro-Diagnostica, Malmö, Sweden). A level of >25 units/ml was interpreted as being positive according to instructions in the kit and as confirmed by validation at the Clinical Immunology laboratory at Uppsala University Hospital, Sweden.

### Radiographs

Radiographs were scored by an experienced investigator (MCW) according to the Larsen method [27] as previously described [28] and documented using the X-Ray RheumaCoach software [29]. Information on change in Larsen score was obtained by subtracting the baseline Larsen score from the score at 1 year or the score at 2 years or by subtracting the Larsen score at 1 year from the score at 2 years.

### HLA analysis

2-digit *HLA-*DRB1 typing was conducted using sequence-specific primer polymerase chain reaction (SSP-PCR) (DR low-resolution kit (2-digit); Olerup SSP, Saltsjöbaden, Sweden) and the PCR products were loaded into 2% agarose gels. An interpretation table was used to determine the specific genotype according to the manufacturers' instructions. *HLA*-DRB1 SE alleles were defined as *01 (except *0103), *04 and *10.

### qPCR

TaqMan Copy Number Assays (Hs02575461_cn) from Applied Biosystems was used to measure *GSTM1* CNV (qPCR). The method was performed using the 7900-HT real-time PCR machine in a 10 ul reaction volume using a 384 well plate containing 10 ng dry DNA, 5 ul TaqMan® Universal PCR Master Mix, No UNG, 0.5 ul of the CNV assay solution, 0.5 ul of the reference assay solution (Applied Biosystems) and 4 ul H_2_0. The qPCR was done using the following cycling conditions: absolute quantification, 95°C for 10 min hold and 40 cycles of 95°C for 15 sec and 60°C for 60 sec. Each individual was represented in quadruplicates on each plate, where at least one sample with known copy number of *GSTM1* was included. A complete description of the work flow can be downloaded from Applied Biosystems website (https://products.appliedbiosystems.com/ab/en/US/adirect/ab?cmd=catNavigate2&catID=606182&tab=Literature); Quick Reference Card, TaqMan Copy Number Assays. The results were analyzed using the software CopyCaller v1.0 downloadable from www.appliedbiosystems.com.

### PCR

In addition to qPCR, we utilized generic PCR as a quality control for detection of *GSTM1* CNV in genomic DNA (1 ul DNA, 30 ng/ul). *GSTM1* primers, forward 5′-CTG GAT TGT AGC AGA TCA TGC-3′, reverse 5′-CTC CTG ATG ATG ACA GAA GCC-3′; housekeeping gene *β-globulin* primers, forward 5′-CAA CTT CAT CCA CGT TCA CC-3′, reverse 5′-GAA GAG CCA AGG ACA GGT AC-3′ [14]. PCR cycling conditions 94°C initial denaturation 15 min., 94°C 30 sec, 63°C 30 sec and 72°C 30 sec for 30 cycles, extra elongation 72°C, 5 min. 60 samples with 0 and 1 copies were randomly selected and used for quality control with 100% match between the TaqMan CNV assay and the generic PCR.

### Statistical analyses

In order to investigate the association between different exposures and risk of developing RA, odds ratios (OR) together with 95% confidence intervals (CI) were calculated. Chi-square test was used for smoking and SE analyses and the non-parametric Kruskal-Wallis test was used when studying DAS28 and Larsen score (JMP 8.0.1).
